# Age-Specific Body Shape Characteristics in the Onset of Spontaneous Pneumothorax: A Comparison Between Teens and 20s

**DOI:** 10.7759/cureus.71922

**Published:** 2024-10-20

**Authors:** Ryo Nonomura, Takafumi Sugawara, Ryuga Yabe, Yutaka Oshima, Takanobu Sasaki, Naoya Ishibashi

**Affiliations:** 1 Thoracic Surgery, Tohoku Medical and Pharmaceutical University, Sendai, JPN; 2 Thoracic Surgery, Tohoku Medical and Pharmaceutical University Hospital, Sendai, JPN

**Keywords:** body shape, bulla, juvenile, physical growth, spontaneous pneumothorax

## Abstract

Background: Primary spontaneous pneumothorax (PSP) has been the subject of many studies, but its pathogenesis remains unclear. Most juvenile PSPs have a tall stature, thin build, and flat thorax, which is described as a "pneumothorax body shape." In this study, we compared the body shapes of spontaneous pneumothorax (SP) patients in their teens and 20s and examined the characteristics of SP patients.

Methods: Among PSPs operated on in our hospital, we compared the body shapes of males in their teens and 20s. In addition to height, weight, and body mass index (BMI), we measured the size of the thorax using computed tomography (CT) and analyzed the relationship between the two groups and height.

Results: A total of 77 cases of PSP in teens and 39 cases of PSP in 20s were entered into the study. Teenage PSPs had significantly lower BMI and were slimmer than those in their 20s. However, there were no differences in thorax size and height other than transverse diameter (TD). Furthermore, almost all thorax sizes of PSPs in their 20s were positively correlated with height, whereas those of PSPs in their teens were not correlated except for TD and vertical length.

Conclusion: The study highlights significant differences in body shape characteristics between teenagers and individuals in their 20s at the onset of SP. These findings emphasize the need for age-specific considerations in the clinical approach to this condition. Further research is required to understand the underlying mechanisms and to optimize treatment strategies accordingly.

## Introduction

Although there are approximately 14,000 surgeries for spontaneous pneumothorax (SP) in Japan [1], the pathogenesis of primary spontaneous pneumothorax (PSP) remains unresolved. We believe that two events, "bulla formation" and "bulla rupture," are involved in the development of PSP. Although both mechanisms are still unclear, the prevailing theory is that bulla formation is related to body shape. This body shape is particularly characteristic of SP patients in their teens and is described as the "SP body shape." The characteristics of the SP body shape are "tall," "thin," and "flat thorax" [2,3]. However, there is no clear definition of these characteristics, and they are merely subjective evaluations by the medical personnel who examine SP patients and are, so to speak, images.

Studies on the body shape of SP have been reported for a long time, although they are not sufficient. Peters et al. [4] reported in 1987 that the vertical length of the thorax was significantly longer in relation to height in patients with SP compared to normal subjects using chest X-rays. Park et al. [5] measured the transverse, anteroposterior, and vertical diameters of the thorax using chest X-rays and chest computed tomography (CT) and compared the ratios of these to height. In addition to PSP, a relationship between SP and funnel chest [6] and Marfan's syndrome [7] with a characteristic thorax has been reported, suggesting a close relationship between SP and the thorax.

Previous studies have suggested an association not only with body size at the onset of SP but also with growth and development, the teenage years being significantly related, especially vertical changes in the thorax being reported to be associated with SP onset [8]. In a report by Fujino et al. focusing on the growth and development of SP cases, both height and weight differed from the norm after the age of 11 years, with height being higher than the norm and weight being lighter than the norm [9]. This report suggests that growth and development at a certain age may have some influence on the development of SP. Furthermore, Mitani et al. reported that asymptomatic SP was more frequent in patients with a higher annual growth rate [10]. Based on these previous studies, it is expected that patients with SP have a characteristic body shape. In our experience, not all patients with SP have the same "SP body shape," but the age of the patients may vary. In our previous studies [11], teenage patients with SP had a history of two or more episodes of SP compared to patients in their 20s or older. Teenage patients with SP may have a tendency to repeat pneumothorax. Most of the previous studies evaluated several age groups together as young adults, and the differences in body shape between the age groups are unknown. In this study, we compared the body shapes of SP patients in their teens and 20s, the most common age group for SP [12], to explore clues to the mechanism of SP development revealed by the differences in body shape.

## Materials and methods

Patients

This cross-sectional study was approved by the Clinical Research Review Committee, Tohoku Medical and Pharmaceutical University Hospital (IRB: 2022-2-074). 

This study included male PSP patients, nonsmokers, in their teens and 20s, who underwent surgery at our hospital from 2011 to 2022. Females were excluded because the biological differences in growth and development between males and females prevented the same evaluation, and because the number of females with PSP was so small that it was deemed unacceptable for analysis. All cases except females and smokers were included in the study.

Methods 　

**Figure 1:**
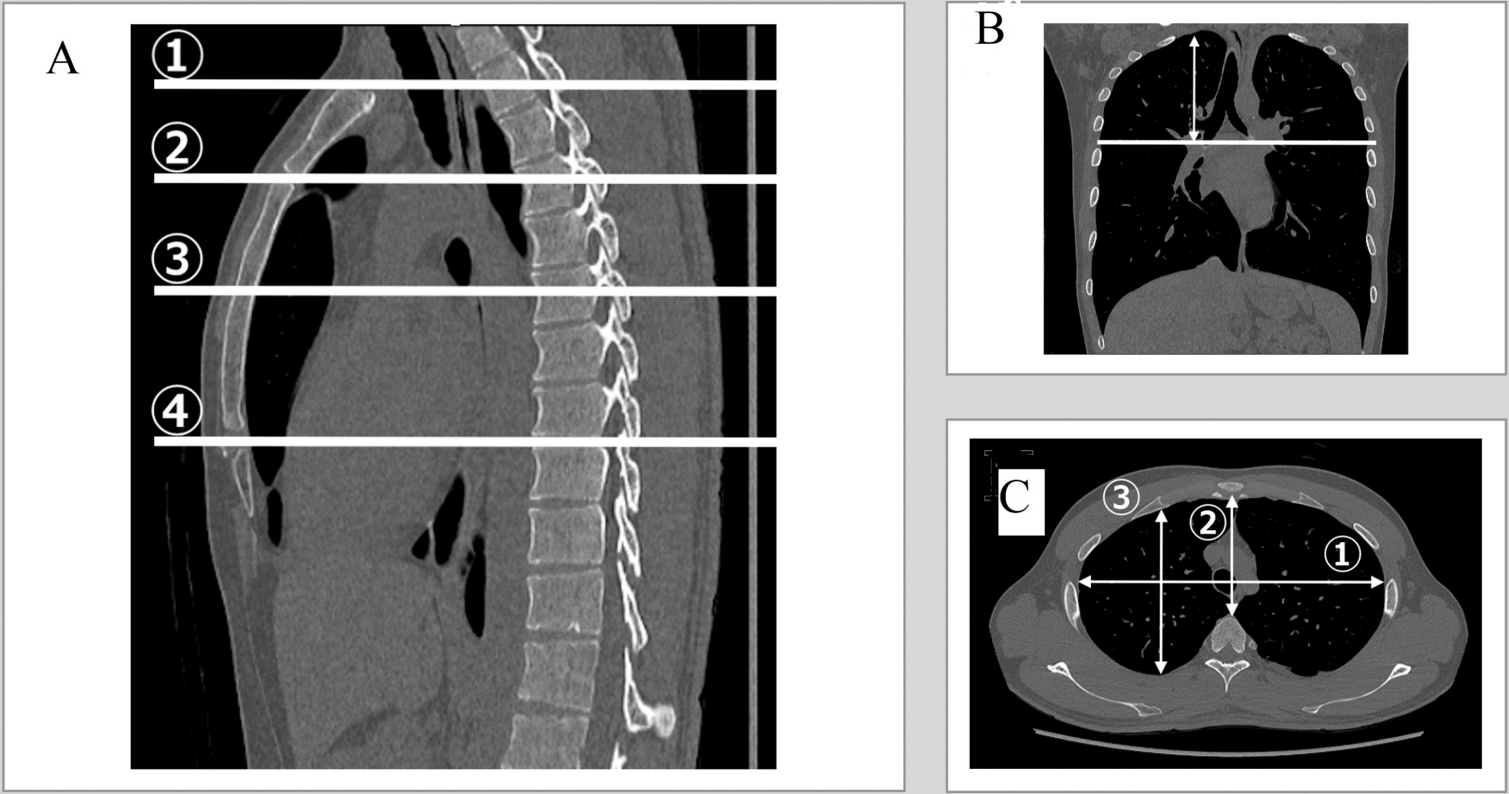
Thoracic measurement using CT (A) Sagittal section. Four heights were specified in the horizontal section. ① manubrium, ② sternal angle, ③ midpoint of sternal body, and ④ xiphi-sternal angle. (B) Horizontal section. Transverse diameter ①, sagittal diameter ②, and right and left anteroposterior diameters ③ were measured in horizontal section. The sagittal diameter was defined as the length from the back surface of the sternum to the anterior margin of the vertebral body. (C) Coronary section. The length from each of the four levels to the apex of the thorax was measured

**Figure 2:**
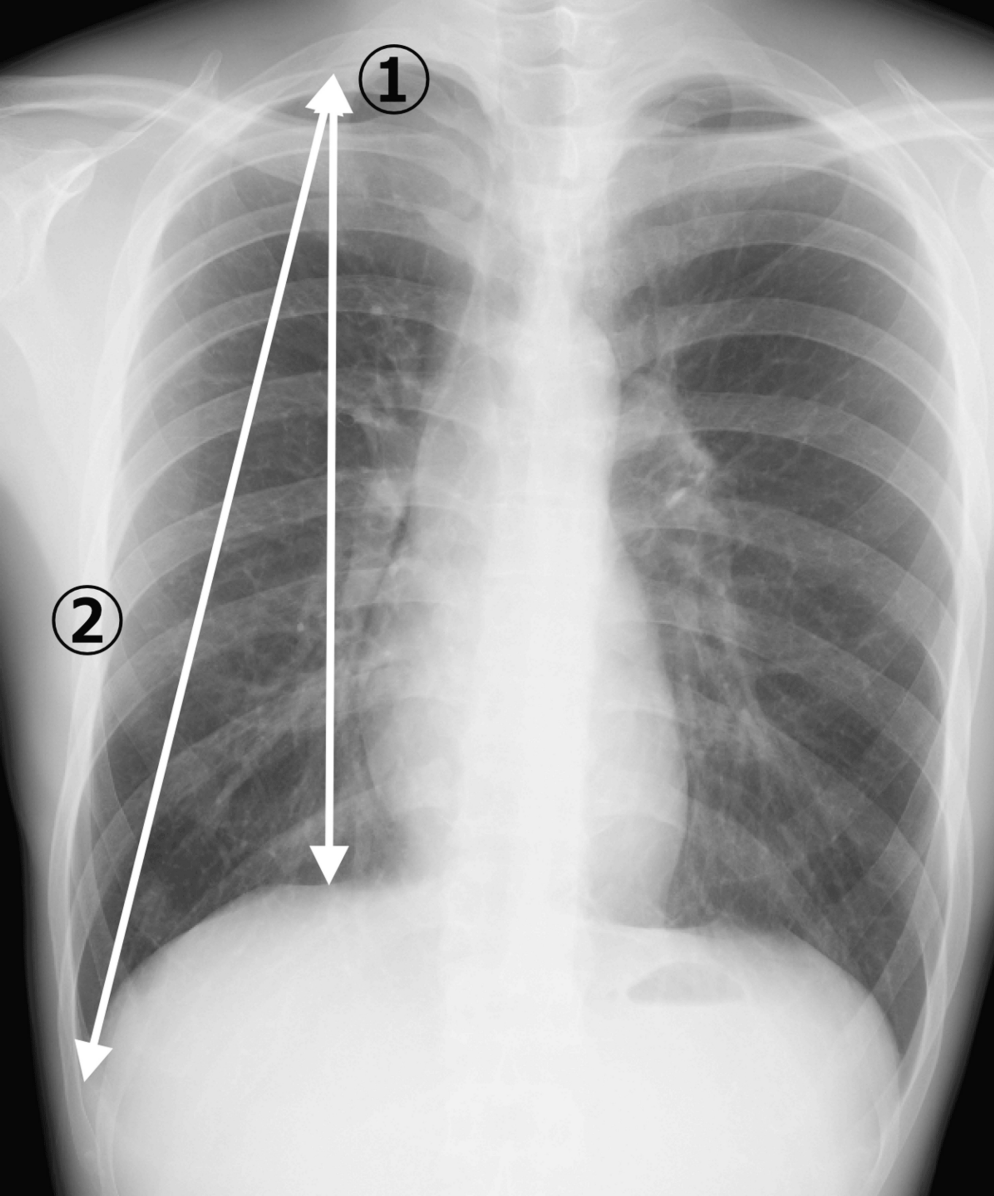
Vertical thoracic length measurement The length from the apex of the thorax to just above the diaphragm ① and from the apex of the thorax to the diaphragmatic angle of the ribs ② were measured using simple chest X-rays

Height, weight, BMI, and thorax size were compared between the PSP group in the teens and the PSP group in the 20s.　Height and weight were extracted from the medical records, and BMI was calculated. The thorax size was measured as follows. CT images taken routinely before surgery were used to determine the size of the thorax. First, the levels of the manubrium, sternal angle, midpoint of the sternal body, and xiphi-sternal angle were defined in the sagittal section (Figure 1A). The height from each level to the apex of the thorax was also measured (Figure 1B). The TD (Figure 1B ①), sagittal diameter (SD) (from the back of the sternum to the anterior margin of the vertebral body) (Figure 1C ②), and anteroposterior diameter of the thorax were measured at the four levels (Figure 1C ③). Vertical lengths were measured from the apex of the thorax to just above the diaphragm (Figure 2 ①) and from the apex of the thorax to the diaphragmatic angle of the ribs (Figure 2 ②) using chest X-rays.

A high-resolution CT (Aquilion 64, One, Prime SP, Toshiba) was used for chest CT imaging. Images were taken from the apex to the base of the lungs during full inspiration in the supine position in helical mode. The CT scan parameters were as follows: matrix size, 512 x 512; slice thickness, 1 mm.

Statistical analysis

All continuous variables were tested for normality using the Kolmogorov-Smirnov test. For between-group comparisons, variables following a normal distribution were compared using the t-test for means. In contrast, those not following a normal distribution were compared using the Mann-Whitney U test for medians. The relationship between height and thoracic parameters was shown using Spearman's correlation coefficient, and a correlation coefficient (|γ：correlation coefficient|) > 0.3 and p-values < 0.05 were considered a significant correlation.

## Results

A total of 116 patients (77 in the PSP group in their teens and 39 in the PSP group in their 20s) were included in the study. The patient background is shown in Table 1. The median age, height, weight, and BMI in the PSP group in their teens and the PSP group in their 20s were as follows: age, 17.0 (16.0-18.0) years vs. 21.0 (20.0-24.5) years (p < 0.0001); height, 173.7 (168.2-175.3) cm vs. 174.9 (170.8-181.2) cm (p = 0.0614); weight 52.5 ± 5.8 kg vs. 58.9 ± 9.7 kg (p < 0.001); and BMI, 17.6 (16.5-18.7) vs. 18.9 (17.9-20.5) (p < 0.001).　

**Table 1:** Patient background and thorax size by age HT: Height; BW: body weight; BMI: body mass index; TD: transverse diameter;  SD: sagittal diameter; RAPD: right anterior-posterior diameter; LAPD: left anterior-posterior diameter

Factor		Teens (n = 77)	20s (n = 39)	p-value
	HT	173.7 (168.2-175.3)	174.9 (170.8-181.2)	0.061
	BW	52.5 ± 5.8	58.9 ± 9.7	<0.001
	BMI	17.6 (16.50-18.7)	18.9 (17.9-20.5)	<0.001
Manubrium				
	TD	193.9 ± 20.4	200.9 ± 18.9	0.073
	SD	41.1 (35.9-48.0)	46.3 (40.9-49.7)	0.059
	RAPD	75.3 ± 11.6	74.7 ± 13.3	0.787
	LAPD	79.2 ± 11.2	79.6 ± 10.7	0.824
Sternal angle				
	TD	236.7 ± 11.6	241.4 ± 13.1	0.051
	SD	70.0 (64.6-76.3)	69.6 (64.2-79.3)	0.928
	RAPD	121.8 ± 11.3	120.6 ± 10.6	0.601
	LAPD	124.8 ± 10.2	124.6 ± 10.4	0.915
Midpoint of the sternal body				
	TD	247.5 (240.32-254.0)	255.0 (246.17-260.37)	0.003
	SD	88.1 ± 12.2	86.2 ± 12.7	0.442
	RAPD	141.3 (131.1-149.3)	140.6 (131.8-152.7)	0.944
	LAPD	143.7 ± 11.1	1143.1 ± 13.6	0.793
Xiphi-sternal angle				
	TD	242.5 (234.3-250.9)	249.2 (245.3-259.7)	<0.001
	SD	88.9 ± 14.0	86.5 ± 13.4	0.374
	RAPD	144.9 ± 12.8	142.4 ± 18.6	0.393
	LAPD	146.0 (137.5-154.5)	144.7 (133.3-155.8)	0.790

The results of the TD, SD, right anteroposterior diameter (RAPD), and left anteroposterior diameter (LAPD) measured by the method shown in Figures 1-2 are shown in Table 1 by age group. Next, Table 2 summarizes the relationship between height and each thoracic component by age group.

**Table 2:** Relationship to height by age TD: Transverse diameter; SD: sagittal diameter; RAPD: right anterior-posterior diameter; LAPD: left anterior-posterior diameter; R: right side; L: left side; γ: correlation coefficient

		Teens (n = 77)		20s (n = 39)	
		γ	P-value	γ	p-value
Manubrium					
	TD	0.351	0.00177	0.567	<0.001
	SD	-0.0193	0.868	0.548	<0.001
	RAPD	0.00694	0.952	0.481	0.002
	LAPD	0.163	0.157	0.596	<0.001
Sternal angle					
	TD	0.458	0.000028	0.559	<0.001
	SD	-0.00152	0.99	0.318	0.049
	RAPD	0.129	0.262	0.434	0.006
	LAPD	0.235	0.0393	0.498	0.001
Midpoint of the sternal body					
	TD	0.456	0.0000307	0.375	0.0185
	SD	0.0252	0.827	0.288	0.0754
	RAPD	0.114	0.323	0.448	0.004
	LAPD	0.0937	0.417	0.403	0.0110
Xiphoid process					
	TD	0.287	0.0114	0.339	0.035
	SD	-0.0889	0.442	0.279	0.085
	RAPD	0.111	0.338	0.347	0.030
	LAPD	0.0472	0.683	0.266	0.1020
Vertical direction					
	Apex of thorax 〜 just above diaphragm (R)	0.603	<0.00001	0.478	0.002
	Apex of thorax 〜 just above diaphragm (L)	0.549	<0.00001	0.421	0.008
	Apex of thorax 〜 diaphragmatic angle of ribs (R)	0.544	<0.00001	0.596	<0.001
	Apex of thorax 〜 diaphragmatic angle of ribs (L)	0.49	<0.00001	0.407	0.010
	Apex of thorax 〜 sternal C (R)	0.192	0.0938	0.153	0.351
	Apex of thorax 〜 sternal C (L)	0.32	0.00451	0.249	0.127
	Apex of thorax 〜 sternal angle (R)	0.387	0.000498	0.305	0.059
	Apex of thorax 〜 sternal angle (L)	0.456	0.0000312	0.342	0.033
	Apex of thorax 〜 midpoint of sternal body (R)	0.38	0.000649	0.502	0.001
	Apex of thorax 〜 midpoint of sternal body (L)	0.5	<0.00001	0.499	0.001
	Apex of thorax 〜 xiphoid process (R)	0.324	0.00404	0.559	<0.001
	Apex of thorax 〜 xiphoid process (L)	0.429	0.0001	0.507	<0.001

## Discussion

In Japan, about 14,000 surgeries for SP are performed annually. PSP accounts for 71% of these cases [1]. Despite the large number of cases, the pathogenesis of SP remains unclear. For SP to occur, two events are necessary: the development of a bulla and its rupture. Although both mechanisms are still unclear, several reports suggest [8-10] that the development of a bulla is related to the patient's body shape. This is because young patients with SP often have a characteristic body shape. In Japan, this is called the SP body shape, which refers to a tall, thin body with a flat thorax. The term "thin" does not refer to a body shape change with pathological weight loss but rather to a body shape with no nutritional or muscle mass problems. However, all of these characteristics are abstract and do not precisely define the SP body type [2,10].

Casha et al. compared a group of patients with SP to an age-matched control group and found that their thoraxes were both vertically and laterally elongated and flattened. The thoraxes of the SP patients were longer and flatter than those of the controls. Additionally, they examined pleural stress, reporting that the apex of the lung exerted 20 times more force than the other parts of the lung [13]. Fujino et al. and Chang et al. concurred with these vertical characteristics [8,9]. Akkas et al. also developed a prediction model for SP using a novel score based on the vertical length of the thorax and BMI [14]. Numerous studies have examined the distinctive body shape associated with SP. As demonstrated by Noh et al., there is a significant difference in body size and surgical outcomes between patients in their early teens (under 16) and those over 19 years of age [15]. These findings suggest that the characteristics of PSP are not uniform. SP patients in their teens also report a lower BMI than those in their 20s [15,16]. However, no detailed comparison of body size differences between SP patients in their teens and 20s has been found to date, including other studies.

The most notable result of this study is the relationship between thorax size and height. Surprisingly, we could not find any studies that examined this relationship. While the 20s group showed positive correlations between height and most thoracic measurement factors, including vertical length, the teens group showed positive correlations only for transverse diameter and vertical length, with a weak connection between anterior-posterior diameter and height growth. This suggests that the sagittal and anteroposterior diameters of teenage SP patients may be changing, different from lateral or longitudinal growth. It is assumed that the sagittal and anteroposterior diameters of the thorax do not keep pace with overall skeletal growth, resulting in a flat and vertically elongated thorax.

Teenagers are at higher risk of neo-bulla than patients over 20 years old (age < 20 vs. age ≥ 20: 44.8 vs. 8.2%) [16]. The immature lung tissue of teenagers may be excessively affected by surgical invasion [16,17]. Furthermore, patients with characteristic thoracic changes may neoplasticize the bulla. Tsuboshima et al. stated that for this reason, it may be necessary to delay surgical treatment of teenage patients with SP [16].

Limitations of this study and future directions include the need for comparison with normal subjects. Since only SP cases were included, it is possible that the characteristic body shape of the teenage SP patients identified in this study is simply the normal body shape during the growth stage. Future research should compare the physical characteristics of SP patients with those of normal teenagers to further explore these physical characteristics. In addition, it will be important in future studies to consider sex, race, national, and regional differences that may influence body shape.

## Conclusions

Teenage patients with SP were found to have a thorax that differs from those in their 20s, particularly in relation to height. Further validation is needed to determine whether the findings of this study are specific to teenage SP patients.
